# Clinical and biochemical characterization of hepatitis B surface antigen-positive patients with or without *Helicobacter pylori* co-infection at the University of Gondar Comprehensive Specialized Hospital, Northwest Ethiopia

**DOI:** 10.3389/fimmu.2025.1553411

**Published:** 2025-06-18

**Authors:** Mekuriaw Belayneh, Bantayehu Addis Tegegne, Mulualem Lemma, Zenahebezu Abay, Yeshambel Belyhun

**Affiliations:** ^1^ Department of Medical Laboratory Sciences, College of Medicine and Health Sciences, Debremarkos University, Debre Markos, Ethiopia; ^2^ Department of Pharmacy, College of Medicine and Health Sciences, Debremarkos University, Debre Markos, Ethiopia; ^3^ Department of Biomedical and Laboratory Sciences, College of Medicine and Health Sciences, University of Gondar, Gondar, Ethiopia; ^4^ Department of Internal Medicine, College of Medicine and Health Sciences, University of Gondar, Gondar, Ethiopia

**Keywords:** HBV, chronic liver disease, H. pylori, HBV/H. pylori co-infection, Ethiopia

## Abstract

**Background:**

Hepatitis B virus (HBV) is a major global public health issue and the most common etiology of chronic liver disease (CLD). The relationship between *Helicobacter pylori* and HBsAg+ patients was not well investigated and has attracted much scientific and clinical interest, although the relationship remains controversial.

**Objective:**

This study aimed to assess the clinical and biochemical characteristics of HBsAg+ liver disease patients with and without *H. pylori* infection.

**Methods:**

From April 1, 2021, to March 30, 2022, a hospital-based cross-sectional study was done at the University of Gondar Comprehensive Specialized Hospital on 384 known HBsAg+ liver disease patients recruited using a convenient sampling technique. All the HBsAg+ patients were tested for fecal *H. pylori* antigen, and blood specimens were analyzed for ALT, AST, ALP, ALB, TP, BILT, TG, and TChol tests using an automated biochemistry analyzer. GraphPad Prism 8.02 and SPSS 25 were used for data analysis, considering a statistically significant *P-value* of 0.05.

**Results:**

*H. pylori* co-infection was found in 153 (39.8%) of HBsAg+ study participants. ALT, AST, and total cholesterol mean levels were significantly higher in patients co-infected with *H. pylori* (p<0.04). Portal hypertension (47.8%), variceal bleeding (60.7%), and hepatocellular carcinoma (HCC) (57.5%) were more common (p< 0.01) in patients with HBV and H. pylori co-infection.

**Conclusions:**

ALT, AST, and TChol mean levels were higher in *H. pylori* co-infected HBsAg+ patients. Our findings showed that *H. pylori* has a role in the elevation of clinical and biochemical parameters in HBsAg+ liver diseases.

## Introduction

Hepatitis B virus (HBV) is a partially double-stranded hepatotropic DNA virus (1). It is an enveloped DNA virus that belongs to the family *Hepadnaviridae*, genus *Orthohepadnavirus* (2). The envelope surrounds an icosahedral nucleocapsid, which encloses a partially double-stranded, relaxed circular DNA (rcDNA) genome of ~3.2 kilobases (3). Four partially overlapping open reading frames (ORFs), termed P (polymerase), S (surface), C (core), and X (HBx protein), define the coding capacity of the HBV genome (4). Mutations in the HBV genome have been observed in all four ORFs in both acute and chronic HBV-infected individuals (5). A total of ten different genotypes of HBV (A-J) have been reported worldwide (6).

Acute HBV infection in healthy and immune-competent adults causes self-restrictive disease with less than 2-3% progression to serum hepatitis B surface antigen-positive (HBsAg +) chronic hepatitis B (CHB) infections (7). The reason behind the adaptive replication of HBV in hepatocytes is mainly due to a lack of functional innate DNA sensing pathways to recognize, control, and clear the virus (8). The adaptive immune response mediates both viral clearance and liver damage, but HBV appears to cause little or no innate immune activation (9). Both the inability of the immune system to resolve CHB and the unique replication strategy of HBV to form a stable covalently closed circular DNA (cccDNA) minichromosome in the hepatocyte nucleus enable infection persistence (10).

The natural course of CHB infection can be divided into four chronological phases based on virus-host interactions: The immune-tolerance phase is characterized by active replication of HBV, HBV e antigen (HBeAg) positivity, and normal alanine transferase (ALT) levels. In the immune clearance phase, HBeAg-positive patients have elevated serum ALT levels and fluctuating HBV-DNA levels. The third stage is the inactive carrier state, in which patients clear HBeAg and develop the corresponding antibody to HBeAg (HBeAg seroconversion), with the remission of liver disease (11). Approximately 20 −30% of individuals in the inactive carrier state may experience a viral relapse and enter the reactivation phase (fourth stage) during follow-up (12, 13).

HBV is not directly cytopathic for hepatocytes. Unlike other viruses, such as hepatitis C virus (HCV) or human immunodeficiency virus (HIV), which enter a rapid phase of propagation after infection, acute HBV infection is preceded by low HBV DNA and antigens in serum and liver for many weeks before the subsequent amplification and spreading phase of HBV infection (14). The virus adapts to different mechanisms for persistent infection and is thought to be a ‘stealth virus’, which poorly induces the expression of type 1 interferon (15).

Liver cirrhosis is a diffuse condition characterized by fibrosis and nodule formation, with CHB virus infection as one of the causes (16). The dysfunctional immune responses play an essential role in persistent HBV infection as well as liver inflammation (17). When comparing the characteristics of immune responses in acute and chronic hepatitis B, Chronic HBV infection develops due to the failure of HBV-specific immune responses (18). Chronic infection of HBV revealed impaired dendritic cell (DC) function, which is reflected by the weakness of both T-cell and B-cell virus-specific immune responses in CHB patients (19).

Persistent exposure of T cells to HBV antigens is crucial for maintaining depressed T cell functionality (20). The quantitative and functional deficiencies of the HBV-specific T-cell response are well-acknowledged as a primary contributor to viral persistence (21). The HBV-specific T cell response is modulated during HBV infection by multi-factorial mechanisms, including programmed cell death 1 (PD-1) expression, IL-10, arginase, myeloid suppressor cells, and T regulatory cells (22).

The humoral immune responses will hand over the virus after release from hepatocytes (23). The released antigens (HBsAg, HBeAg, and HBcAg) will induce the production of their respective antibodies. Hepatitis B core antigen (HBcAg) will not circulate in the blood as it is found in the core of the virus. Anti-HBc antibodies are used to differentiate between acute and chronic HBV infections (24).

*H. pylori* causes both active and chronic infection and can lead to several disorders, from chronic gastritis to gastric adenocarcinoma, and activates both innate and adaptive immune responses, but the response fails to eradicate the infection (25). Although it is believed that *H. pylori* is a type of ‘commensal bacterium”, it cannot be classified as normal flora because all patients with gastro-duodenal *H. pylori* colonization show histological gastroenteritis (26). To complete the colonization process and cause harm to the gastric mucosa, the bacterium must overcome the stomach acid barrier and infiltrate the mucus layer (27). Gastric epithelial cells (GECs) are a primary target for *H. pylori* infection and actively contribute to the development of acute and chronic inflammation (28).

*H. pylori* may induce immunosuppressive Tregs, dampening antiviral immune responses against HBV and facilitating viral persistence. Chronic *H. pylori* infection could also exacerbate T-cell exhaustion, reducing control of HBV replication and increasing liver damage (29, 30). The greatest feature of *H. pylori* is its ability to last for years in the gastric epithelium of the host (31). This adaptive property occurs due to a complex mechanism of *H. pylori* persistence mediated by proteins, glycoconjugates, and lipids exposed on the surface of this bacterium (32). Gastric innate immune effectors can either eliminate the bacteria or mobilize adaptive immune responses (e.g., Toll-like receptors (TLRs) and cytosolic DNA sensor/adaptor proteins) (33). Recent studies have shown that *H. pylori* infection most often results in M1 (Inflammatory) and Mreg (Regulatory) macrophage activation (34). The recruited macrophages at the site of infection can produce IL-12, which stimulates T-helper 1(Th1) cells and the production of cytokines such as IFN-γ (35).

*H. pylori* escapes identification by pattern recognition receptors (PRR) by multiple methods, including avoidance of recognition by TLRs and inhibition of c-type lectin and Dendritic cell-specific intercellular adhesion molecule 3-grabbing nonintegrin (DC-SIGN) mediated signaling (36). Although the acquired immune response to *H. pylori* is composed of both Th1- and Th2-type cells, cytokine profiles indicate a predominance of a Th1 response (37). Although *H. pylori* is an extracellular pathogen, its immune response is biased to the Th1 type (38).

In recent years, *H. pylori* has been reported to be associated with the development of a variety of extra-digestive manifestations, including type 2 diabetes, and cardiovascular and liver diseases (39). Chronic *H. pylori* infection induces a Th1-mediated inflammatory response, potentially contributing to systemic inflammation that could exacerbate liver injury. Virulence factors (e.g., CagA, VacA) may enter the bloodstream, promoting oxidative stress and pro-inflammatory cytokine release, which might accelerate liver fibrosis or carcinogenesis. This study tried to evaluate the burden and clinical contribution of *H. pylori* infection on the outcome of HBV-related liver diseases in Northwest Ethiopia.

The relationship between *H. pylori* and liver diseases remains controversial. Recent studies have been trying to investigate the association of *H. pylori* in the progression of diseases other than gastrointestinal diseases (40). The presence of *Helicobacter* sp*ecies* has been reported in the hepatic tissues of patients with different hepatic disorders. Chronic inflammation from H. pylori might promote fibrosis progression to cirrhosis and HCC. However, confounders like viral hepatitis or environmental toxins complicate these associations (41). *H. pylori* has been implicated in some extra-digestive diseases such as cardiovascular, neurologic, and hepatobiliary conditions (42). *H. pylori* has the induction potential of regulatory B cells, suggesting its role in diminishing the response of effector B cells during HBV infections (43).

*H. pylori* infection in patients with liver cirrhosis may impact the exacerbation of inflammatory injuries in the stomach, which could directly or indirectly lead to a deficiency of liver function (44). Research conducted in China also showed that the *H. pylori* positivity rate is high among patients with CHB, and *H. pylori* is a putative risk factor in the development of HBV-related complications. This is because *H. pylori* reaches the liver via the bloodstream or the biliary system and then becomes an independent etiological factor causing inflammation (45).

In *H. pylori*-infected individuals, a Th2 cell-related response induces IgG1 production while a Th1-related response contributes to IgG2 production through IL-2 and IFN-γ secretion (46). *Helicobacter* species are regarded as important in the development of hepatobiliary illnesses because they can produce inflammatory, fibrotic, and necrotic damage to the liver, which can progress to HCC (47). In the present study, we tried to assess the interplay of HBV and *H. pylori* infections on CHB-related liver disease progression.

## Materials and methods

### Study area, design, and period

The study was conducted at the University of Gondar Comprehensive Specialized Hospital, located in Gondar city, Amhara National Regional State, Northwest Ethiopia. Gondar City is 738km Northwest of Addis Ababa, the capital city of Ethiopia. Based on the Central Statistical Agency of Ethiopia (CSA), Gondar City had a total population of 500,788, of whom 300,000 were men and 200,788 were women (48). The University of Gondar Comprehensive Specialized Hospital is a tertiary care center serving approximately 7 million people in Northwest Ethiopia. A hospital-based cross-sectional study was conducted from April 1, 2021, to March 30, 2022. It was a single-center institution-based study.

#### Source and study populations

All HBsAg+ liver disease patients attending the University of Gondar Comprehensive Specialized Hospital. HBsAg+ patients presented at the University of Gondar Comprehensive Specialized Hospital during the study period and were eligible to study.

#### Inclusion criteria exclusion criteria

Patients who were positive for HBsAg and with the age of above 18 years old were included in the current study. We excluded patients with clinically confirmed schistosomiasis, and viral hepatitis other than HBV, HIV, and pregnancy.

### Sample size and sampling technique

The sample size was determined using the single population proportion formula by considering the prevalence of *H. pylori* in HBsAg+ patients =50% (p=0.5), Z α /2 = 1.96, and margin of error =5% (d=0.05). Finally, the total sample size became 384 HBsAg+ individuals who were recruited using a convenient sampling technique.

### Data collection and laboratory methods

#### Demographic and clinical data collection

Demographic and clinical data were collected following the approval of the study protocol by the ethical review committee of the University of Gondar. At each data collection unit, trained nurses collected all relevant information (demographic, clinical) using a structured questionnaire in a face-to-face interview with the patient and from the patient’s medical records. During clinical data collection, the patients were enrolled from the inpatient and outpatient departments of the gastroenterology clinic at the University of Gondar Comprehensive Specialized Hospital. The clinical parameters, such as portal hypertension, hepatocellular carcinoma (HCC), variceal bleeding, and hepatic decompensation, were defined based on the radiological (ultrasound, endoscopy/colonoscopy) and pathology (biopsy) findings.

#### Specimen collection and processing

We collected about eight milliliters (8 mL) of venous blood by serum separator tubes (SST) from the forearms of all eligible patients. The serum was isolated from the collected blood samples by centrifuging at 3500 rpm for 5 minutes in the serology laboratory and separated into different serum test tubes. The first serum tube (4 ml) was transported to the clinical chemistry lab for biochemical testing. For further serological and virological examination, the second serum was transferred to cryotubes and stored at -80°C.

In addition, a stool sample was collected from each CLD patient included in this study for the identification of the *H. pylori* fecal antigen (RAPID Hp StAR test).

#### Biochemical assays

The mean concentrations of alanine transaminase (ALT), aspartate transaminase (AST), alkaline phosphatase (ALP), serum albumin (ALB), total protein (TP), total bilirubin (BILT), total cholesterol (TChol) and triglycerides (TG) were analyzed by using automated DxC AU 700 Chemiluminescence assay (BECKMAN COULTER, Ireland Inc., Lismeehan, O’Callaghan’s Mills, Co. Clare, Ireland) following the manufacturer’s instructions. Both enzymatic and non-enzymatic assays were performed by the same automated biochemistry analyzer.

### Data quality control

Study site assessment and pretest of the tools and assays were done before data collection to optimize the experimental setup. Data collectors were given appropriate training about data collection and related procedures. Positive and negative controls were run against HBsAg and *H. pylori* tests. Patient samples were collected, shipped, and stored under appropriate conditions and strict supervision. Standard operating procedures (SOPs) and optimization protocols were followed during serological and virological assays.

### Data analysis

Data entry was done via Microsoft Excel 2013. Normally distributed data were expressed as mean ± standard deviation, and skewed data were expressed as median (interquartile range, IQR). Statistical analyses were performed using SPSS 25.0 (IBM, New York) or GraphPad Prism 8.0.2. Categorical variables were analyzed by the Chi-square test, and nonparametric tests (Mann-Whitney test) were used for non-normal continuous data, by taking a *P-value* of<0.05 as statistically significant.

### Ethical considerations

The study protocol was reviewed ethically and approved by the ethical committee of the School of Biomedical and Laboratory Sciences (SBLS) at the University of Gondar. Ethical clearance with a reference number (Ref. No. SBMLS/2759) was obtained. Study participants were asked for their consent to be included in the study. Study participants who tested positive for HBsAg and/or *H. pylori* during screening were linked to healthcare providers of the UoG hospital. All the information obtained from the study participants was coded to maintain confidentiality.

## Results

### Demographic characteristics of the study participants

From a total of 384 HBsAg+ adults enrolled in this study, 153 participants were *H. pylori* co-infected (39.8%). 259 (67.4%) subjects were males, and 268 (69.8%) of them were rural residents. Overall, 47.1% of subjects were unable to read and write, and 221 (57.6%) of them were farmers. Overall, 317 (82.6%) participants were married. Three hundred thirty-nine (88.3%) of participants were not alcohol users (**Table 1**).

**Table 1:** Socio-demographic characteristics of HBsAg+ study participants with and without *H. pylori* at University of Gondar Comprehensive Specialized Hospital, Northwest Ethiopia, 2022.

Characteristics		*H. pylori* status			P-value
		Positive, n (%)	Negative, n (%)	Total, n (%)	Sub-total
Sex	Male	99 (64.7)	160 (69.2)	259 (67.4)	0.02
	Female	54 (35.3)	71 (31.8)	125 (32.6)	
	Ratio (Male/female)	1:8:1	2.25: 1	2.07: 1	
Age (years)	≤ 24	14 (9.1)	32 (13.8)	46 (12)	0.14
	25-30	15 (9.8)	41 (17.7)	56 (14.5)	
	31-34	15 (9.8)	15 (64.9)	30 (7.8)	
	35-39	17 (11.1)	35 (15.1)	52 (13.5)	
	≥40	92 (60.1)	108 (46.7)	200 (52)	
Residence	Rural	113 (73.8)	155 (66.2)	268 (69.8)	0.03
	Urban	40 (26.2)	76 (33.8)	116 (30.2)	
Level of education	Unable to read and write	77 (50.3)	104 (45)	181 (47.1)	0.06
	Read and write	42 (27.4)	74 (32)	116 (30.2)	
	Primary school	14 (9.1)	29 (12.5)	43 (11.2)	
	Secondary school	13 (8.5)	12 (5.2)	25 (6.5)	
	College and above	7 (4.6)	12 (5.2)	19 (4.9)	
Marital status	Married	128 (83.7)	189 (81.8)	317 (82.6)	0.2
	Single	18 (11.7)	28 (12.1)	46 (12)	
	Divorced	5 (3.3)	10 (4.3)	15 (3.9)	
	Widowed	2 (1.3)	4 (1.7)	6 (1.5)	
Occupation	Farmer	93 (60.8)	128 (55.4)	221 (57.6)	
	Merchant	29 (18.9)	53 (22.9)	62 (16)	
	Housewife	12 (7.8)	24 (10.4)	36 (9.4)	0.04
	Employee	12 (7.8)	16 (6.9)	28 (7.3)	
	Student	7 (4.6)	10 (4.3)	17 (4.4)	
Smoking habit	Smoker	7 (4.6)	5 (2.2)	12 (4.4)	0.6
	Non-smoker	146 (95.4)	226 (97.8)	372 (95.6)	
Alcohol use	Ever used	23 (15)	22 (9.5)	45 (11.7)	0.06
	Not used	130 (85)	209 (90.5)	339 (88.3)	
Total		153 (39.8)	231 (60.2)	384 (100)	

### Clinical and biochemical features of study participants

Among 384 HBsAg+ study participants, 83 (21.6%) patients used traditional medicine in different forms or preparations. Regarding their HBV treatment status, 106 (27.6%) patients were treatment-naïve. The treatment status and traditional medicine use of HBsAg+ patients showed no significant differences between the two groups of participants. Portal hypertension (47.8%), variceal bleeding (60.7%), and hepatocellular carcinoma (HCC) (57.5%) had a significant association with *H. pylori* co-infection (p<0.01) (**Table 2**).

**Table 2:** Clinical characteristics of 384 HBsAg+ study participants with and without *H. pylori* at University of Gondar Comprehensive Specialized Hospital, Northwest Ethiopia, 2022.

Characteristics		*H. pylori*+, n (%)	*H. pylori-*, n (%)	Total, n (%)	*P-value*
Ascites	Yes	81 (44.8)	100 (55.2)	181 (47.1)	0.64
	No	72 (35.5)	131 (64.5)	203 (52.9)	
Portal hypertension	Yes	77 (47.8)	84 (52.2)	161 (42.9)	0.007
	No	76 (34.1)	147 (65.9)	223 (57.1)	
Hepatic decompensation	Yes	14 (58.3)	10 (41.7)	24 (6.2)	0.056
	No	139 (38.6)	221 (61.4)	360 (93.8)	
Variceal bleeding	Yes	37 (60.7)	24 (39.3)	61 (15.9)	<0.001
	No	116 (38.7)	207 (61.3)	323 (84.1)	
HCC	Yes	23 (57.5)	17 (42.5)	40 (10.4)	0.01
	No	130 (37.8)	214 (62.2)	344 (89.6)	
Treatment status for HBV	Naïve	39 (36.8)	67 (63.2)	106 (27.6)	0.45
	TDF experienced	114 (41)	164 (59)	278 (72.4)	
Traditional medicine of HBV	Ever used	36 (43.4)	47 (56.6)	83 (21.6)	0.46
	Not used	117 (38.9)	184 (61.1)	301 (78.4)	
Total, N		153 (39.8)	231 (60.2)	384 (100)	

N, Number at each group; n, Number; %, Percentage; TDF, Tenofovir Disoproxil Fumarate; HCC, Hepatocellular Carcinoma; HBV, Hepatitis B virus.

Among the biochemical parameters, ALT, AST, and TChol levels were significantly higher in the *H. pylori co-*infected patient group (p<0.01). Although there was a difference in the mean value of ALP, ALB, TP, and BILT in the two groups, the difference was not statistically significant. Overall, the mean concentrations of liver enzymes and proteins were higher in the *H. pylori* co-infected group of patients (**Table 3**).

**Table 3:** Comparison of Biochemical Tests in 384 HBsAg+ Study Participants with and without *H. pylori* co-infection at University of Gondar Comprehensive Specialized Hospital, Northwest Ethiopia, 2022.

Parameter (U/L)	*H. pylori-* mean levels	*H. pylori* +, mean levels	Reference range	*P-value*
ALT (U/L)	35	40	0-30	0.01
AST (U/L)	42	56	0-37	0.002
ALP (U/L)	104	120	40-106	0.146
ALB (g/dl)	3.7	3.8	3.4-5.4	0.93
TP (g/dl)	7.1	7.2	6-8.3	0.28
BILT (mg/dl)	1.2	1.2	0.1-1.2	0.99
TChol (mg/dl)	130	140	<200	0.04
TG (mg/dl)	92	97	<150	0.06
Total, N	231 (60.2)	153 (39.8)		384 (100)

N, Number at each group; ALT, Alanine transaminase; AST, Aspartate transaminase; ALP, Alkaline phosphatase; ALB, Albumin; TP, Total protein; BILT, Total bilirubin; TChol, Total Cholesterol; TG, Triglycerides. U/L, Unit per liters.

## Discussions

Infections with HBV and *H. pylori* are two major public health issues in Ethiopia. Epidemiological studies in Ethiopia have reported a high prevalence of both infections, but there were no previously published reports on the relationship between HBV and *H. pylori* in liver disease patients and the contribution of *H. pylori* co-infection, particularly in Northwestern Ethiopia. Assessing the clinical and laboratory features of liver disease patients infected with these important pathogens is crucial for understanding and managing the disease progression in HBV-liver disease. In the present study, we tried to characterize the clinical, serological, and virological features of HBsAg+ liver disease patients with and without *H. pylori* co-infection.

In the current study, the prevalence of *H. pylori* infection (39.8%) among HBsAg+ patients appeared lower than the overall pooled prevalence in the general Ethiopian population (52.2%) (49). However, the prevalence of *H. pylori* infection in the current study population was comparable to similar studies on HBV-related liver disease patients (40, 50). Previous studies reported a higher *H. pylori* prevalence in the general population than in liver disease patients. This discrepancy could be due to the repeated exposure of patients to different antibiotics during hospital visits, variations in the sensitivity of fecal *H. pylori* antigen tests, and the time trend of studies showing a decreasing pattern of *H. pylori* infection.

*H. pylori* colonization of the liver may occur through bacterial translocation from the stomach through the portal system, especially in the advanced stages of liver disease when portal hypertension develops. Furthermore, the bacteria can reach the liver via circulating phagocytes and macrophages. The present study was in agreement with previous studies that demonstrated that post-inflammatory liver cirrhosis complications were more frequent in *H. pylori*-infected patients than in the *H. pylori*-negative patient group, as *H. pylori* could upregulate the expression of various inflammatory factors (50–52). In our study population, HBV-induced complications of liver cirrhosis, such as portal hypertension, variceal bleeding, and HCC, were more prevalent in *H. pylori* co-infected patients than in the HBV-mono-infected group of patients. In this study, hepatic decompensation was also higher among the co-infected group, although it showed no significant difference. These clinical findings confirm our hypothesis that *H. pylori* co-infection has a synergistic effect on the progression of CHB-related liver diseases (50).

Liver enzymes and non-enzymatic biochemical markers of liver function had been increased in our study subjects who had *H. pylori* co-infection, in line with similar studies (53). In this study, ALT, AST, and TChol levels were significantly higher in the *H. pylori* co-infected group of patients, while TG and ALP levels were slightly elevated in the same group of patients. Our results support the theory that liver colonization with *H. pylori* bacteria promotes liver function deterioration via toxic injury and autoimmune inflammation. These findings align with studies reporting the role of *H. pylori* in CHB disease progression (54, 55).

There were several limitations in this study. First thing, we had no access to molecular, experimental, and immunological assays. We just used a cross-sectional study design with convenience sampling techniques. We did not use quantitative assays to measure the level of fecal antigen for the diagnosis of *H. pylori* infection. Consequently, the exact change of values might not be revealed in the HBV/*H. pylori* co-infected and HBV mono-infected patients. Saying all these, this study provided insight into the contribution of *H. pylori* infection among HBV-infected liver disease patients.

## Conclusions and recommendations

The ALT, AST, and TChol levels were increased during *H. pylori* co-infection. Our data also showed that clinical parameters, including portal hypertension, variceal bleeding, and hepatocellular carcinoma (HCC), were more frequently detected in HBsAg/*H. pylori* co-infected patients than those with HBsAg mono-infection. From the findings of the current study, it can be concluded that *H. pylori* significantly enhances the clinical, biochemical, and serological parameters during the co-infection of *H. pylori* with HBsAg+ liver disease patients. Physicians who treat liver disease patients are highly recommended to screen and treat patients with *H. pylori* co-infection. We propose active screening for *H. pylori* in patients with CHB. Virological markers of HBV should also be closely monitored during the treatment of CHB-related liver diseases. We would also like to recommend that future researchers conduct large-scale studies that include advanced laboratory methods with an adequate sampling of patients to assess the accurate implications of all test parameters of CHB-related liver diseases co-infected with *H. pylori* bacteria. Besides, it would be imperative that future studies include molecular and immunological methods with a longitudinal cohort of patients and advanced statistical methods.

## Data Availability

The original contributions presented in the study are included in the article/supplementary material. Further inquiries can be directed to the corresponding author.
